# Analysing local-level responses to migration and urban health in Hillbrow: the Johannesburg Migrant Health Forum

**DOI:** 10.1186/s12889-017-4352-2

**Published:** 2017-07-04

**Authors:** Jo Vearey, Kirsten Thomson, Theresa Sommers, Courtenay Sprague

**Affiliations:** 1 African Centre for Migration & Society (ACMS), University of the Witwatersrand, Johannesburg, South Africa; 2 Department of Conflict Resolution, Human Security & Global Governance, University of Massachusetts Boston, Boston, MA USA; 3 Wits Reproductive Health and HIV Institute (WRHI), Faculty of Health Sciences, University of the Witwatersrand, Johannesburg, South Africa; 4 Department of Nursing, College of Nursing & Health Sciences, University of Massachusetts Boston, Boston, MA USA

**Keywords:** Migration, Urban health, Johannesburg, South Africa, Local-level response, City governance, Civil society

## Abstract

Johannesburg is home to a diverse migrant population and a range of urban health challenges. Locally informed and implemented responses to migration and health that are sensitive to the particular needs of diverse migrant groups are urgently required. In the absence of a coordinated response to migration and health in the city, the Johannesburg Migrant Health Forum (MHF) – an unfunded informal working group of civil society actors – was established in 2008. We assess the impact, contributions and challenges of the MHF on the development of local-level responses to migration and urban health in Johannesburg to date. In this Commentary, we draw on data from participant observation in MHF meetings and activities, a review of core MHF documents, and semi-structured interviews conducted with 15 MHF members.

The MHF is contributing to the development of local-level migration and health responses in Johannesburg in three key ways: (1) tracking poor quality or denial of public services to migrants; (2) diverse organisational membership linking the policy process with community experiences; and (3) improving service delivery to migrant clients through participation of diverse service providers and civil society organisations in the Forum. Our findings indicate that the MHF has a vital role to play in supporting the development of appropriate local responses to migration and health in a context of continued – and increasing – migration, and against the backdrop of rising anti-immigrant sentiments.

Johannesburg – a city of just over 4,4 million people – is home to a diverse population of migrants, including internal migrants from elsewhere in the country, migrants from neighbouring southern African nations, and those who have originated from further afield [1]. Known as ‘Egoli’ in isiZulu, or ‘City of Gold’, Johannesburg was historically a destination for those seeking opportunities for work in the mines on the Witwatersrand plateau [2]. Today, thousands continue to travel to the city in search of improved livelihood opportunities. In 2011, almost one third of city residents were born elsewhere in South Africa, and approximately 13% were born outside the country [1, 3]. Whilst reflecting global trends – with cross-border migrants comprising between three and 4% of the South African population [3, 4] – migrants are unevenly distributed across the country. Cities such as Johannesburg, and certain inner-city suburbs, such as Hillbrow, are home to a higher density of internal migrants and non-nationals than other parts of the country [5].

What do these migration patterns mean for the provision and uptake of health services in the inner city? Firstly, while there is evidence of a ‘healthy migrant effect’ – whereby those who migrate tend to have a better health status compared to the host population – and while non-national migrants do not appear to travel to Johannesburg specifically to access healthcare, they may nonetheless require it (see, [4]). Secondly, cross-border migrants without private medical aid or funds to pay for private healthcare are, like the majority of South Africans, reliant on an over-burdened and struggling public healthcare system [6]. Their challenges in accessing public healthcare and other positive determinants of health in Johannesburg, including housing and employment, have been well-documented [4, 7]. Moreover, these challenges can be formidable for migrants who are poor, marginalised or in greatest need of healthcare, such as pregnant women, young children, those with chronic illness and/or mental health needs, and victims of violence [8]. Locally informed and implemented responses that are sensitive to the particular needs of migrant populations are urgently required (for example see, [4, 9]). At national, provincial and local levels in South Africa, however, effective responses to migration and health are lacking [10], in spite of progressive legislation affording non-nationals the right to health services [11].

In the absence of a coordinated response to migration and health in the city, the Johannesburg Migrant Health Forum (MHF) was established in 2008 by the African Centre for Migration and Society (ACMS) at the University of the Witwatersrand, the International Organisation for Migration (IOM) South Africa office, and the Wits Reproductive Health and HIV Institute (Wits RHI). The Johannesburg MHF – an unfunded informal working group – is an example of a civil society driven, local-level response to migration and urban health, which may hold relevance for other settings, given the complex patterns of global migration and population mobility and the pressure to deliver health and social services in diverse contexts [12].

The MHF was set up to enable organisations and individuals working on issues of migration and health in Johannesburg to come together on a regular basis with the aim to *“support member organisations in taking action to address the public health needs of migrants; share knowledge and experience; disseminate research; avoid duplication of effort; and facilitate collaboration where appropriate”* [13]. The need for the MHF was further fostered by the real world challenges of addressing the specific health needs of vulnerable or excluded migrants (undocumented migrants, refugees and asylum seekers), specifically in relation to access to preventative and maternal healthcare, and to medication for HIV and tuberculosis [14]. Populated by members of 15 civil society organisations, the MHF also seeks to hold the state accountable for its constitutional obligation to provide healthcare to non-nationals [13, 15].

The MHF operates as a regular yet informal networking platform, with monthly face-to-face meetings at the Wits RHI offices in Hillbrow and a vibrant online presence in the form of a Facebook page and a Google Group. In September 2016, the latter had 86 members representing a diverse array of non-governmental and community-based organisations, international organisations, academic institutions and researchers. Overall, the complex health needs of cross-border migrants remain the central focus of the Forum. These are tackled through the on-going engagement of Forum members and a deepening of links to research undertaken by and with MHF members [13]. The main activities of the MHF fall into three key areas, spanning research, consultation and support, as outlined in Table 1 below.

**Table 1:** The main activities of the Johannesburg Migrant Health Forum

Key area	Examples of Forum activities
1. Research, education and dissemination	• *Monitoring of public healthcare access challenges* using a monitoring tool to capture the experiences of non-nationals. • *Production of factsheets on migration and health* for journalists and others, answering common questions on migration and health in Johannesburg (Johannesburg Migrant Health Forum, 2015b). • *Production and dissemination of educational materials for migrants, healthcare workers and the general public* in English and French on the rights of non-nationals to access public healthcare within Gauteng Province (Johannesburg Migrant Health Forum, 2014). These materials are freely available electronically for download, printing and dissemination. • *Research presentations* at local policy and research conferences (such as the JHB District Health Research Conference, 2014), on the challenges faced by non-nationals reliant on public healthcare. • *Participation in mental health workshops for refugee community groups and those that support asylum seekers and refugees* (e.g. resource centres police, nurses). These have been led by Lawyers for Human Rights for the United Nations, on addressing the mental health needs of asylum seekers and refugees when accessing services (2014; 2015).
2. Communication and consultation with government	• *Participation in policy consultations* such as the national migration and health consultation co-hosted by the IOM and the ACMS, Wits (2010, 2013) and the annual South African AIDS Conference (via a national working group on migration and HIV). • *Meetings with the Provincial Department of Health* to present data collected by the MHF and research partners on challenges experienced by non-nationals when accessing public healthcare at Provincial Hospitals. Ongoing dialogue to explore possible programmatic solutions (SECTION27, 2015).
3. Support network and knowledge sharing platform	• *Developing support networks* between member organisations and migrant individuals or groups to address specific migration and health challenges experienced in the city, including access to legal representation.

Research has been undertaken in recent years to explore the work and contributions of the MHF to improving migrant access to positive determinants of health, including public healthcare [16, 17]. Here, we report briefly on our experiences as members of the MHF (JV and CS since its inception in 2008; KT since 2011; TS since 2013). We draw on qualitative research conducted by one of us (TS), using in-depth interviews with 15 members of the Johannesburg MHF during 2013 and 2014, participant observation at MHF meetings and a review of MHF meeting documents. Three main findings emerged from this analysis, outlining how the MHF is contributing to the development of local-level migration and health responses in Johannesburg: (1) the Forum acting as a ‘monitoring’ space, tracking poor quality or denial of public services to migrants; (2) diverse organisational membership linking the policy process with community needs and experiences; and (3) participation by diverse service providers and civil society organisations in the Forum as a means of improving overall service delivery to migrant clients. What do these findings tell us about the Forum’s role in generating a local-level response in the City of Johannesburg?

Firstly, the MHF gives members a space and platform for sharing information on local events in the migrant community, as well as local and national policy changes affecting their work and the clients they serve [16, 17]. In this respect, the MHF acts as a ‘monitoring’ system of sorts, able to identify – through the network – specific health clinics and other service providers who provide hassle-free services to migrants [16, 17]. Forum members receive real-time information regarding the quality of care and service received in public facilities in the City. While a formal feedback structure for migrant experiences is currently absent, there is an informal monitoring system where negative experiences of being turned away from facilities or experiencing discrimination may be reported. As noted by one member, *“we see on and off that some nurses are unwilling to work with migrants. We see this more at particular facilities”* (Interview, Legal Services Organisation, Attorney, July 2014). Such information is often shared through the Forum’s online communications channels, such as the email listserv, affording all members the opportunity to incorporate this localised knowledge in their work with migrant clients and *“refer patients to clinics we know to be migrant-friendly”* (Interview, Direct Care Organisation, Clinical Social Worker, July 2014).

Secondly, one of the strengths of the Johannesburg MHF stems from its diversity of membership, which includes migrant-led organisations, academics and researchers, providers of direct clinical care, legal service providers and advocacy organisations. Members are encouraged to bring their expertise and professional contacts to this space, allowing the Forum to draw upon a robust and comprehensive set of stakeholder perspectives, including key expertise in human rights, constitutional law, and health services research and delivery. During interviews with Forum members, views on how the Forum should focus its work were mixed, generating the lively debate that is a hallmark of healthy civil society engagement. Indeed, this debate could be seen as one of the MHF’s greatest strengths. For example, some members felt that the Forum should be used to *“influence policy”* at the local and even national level, while others saw the Forum’s role in terms of supporting direct service delivery to clients (Field Notes, July 2013). One member provided an example of how the diverse areas of expertise among the MHF’s membership might be especially beneficial in the event of an emergency situation: *“[members would] research the issue collectively, consider available options, and come up with the best method to act. Since [our organisation] works with the media, if the consensus was to shed light on the situation, we would draft a press release with input from MHF members, and then disseminate to our media contacts”* (Interview, Advocacy Organisation, Programme Officer, August 2013). Diverse membership also enables the building of operational links between policy processes and community experiences, for example, through collaboration between member organisations that are more visible to local policy makers and member organisations working directly with migrant communities through service provision.

Although MHF members are present in their capacity as individuals [13], their participation in the Forum is a way to feed information and lessons back to their own organisations, thereby improving the ability of these organisations to serve the migrant community. This sentiment was shared by all members who were interviewed. As one participant put it, *“it’s not about us [members of the MHF], but the people we serve. Any method of improving service delivery and assistance to [the migrant] community is what we should do in the Forum”* (Interview, Direct Care Organisation, Programme Manager, June 2013).

In conclusion, the Johannesburg MHF presents an example of a successful, albeit often challenging, local-level, civil society led response to migration and urban health. Important lessons that may be relevant to other settings include the benefit of maintaining an informal, non-binding arrangement within the Forum to allow for the participation of diverse stakeholders. Should such a Forum become formally constituted – for example with a formalised structure, funding and a salaried secretariat – it is likely that some organisations and individuals would no longer be able to participate due to conflicting mandates and concerns relating to representation. Linked to this is the challenge of maintaining a balance between engaging with government officials versus inviting them to have formal involvement in the Forum. As with a shift to a formal constitution, official involvement of government officials in the MHF would change the dynamics of the Forum and create potential conflicts of interest, particularly for members who receive state funding.

Moving forward, the MHF continues to face challenges relating to its operation within a political and social context that is increasingly fuelled by anti-immigrant sentiments and increasing pressures on state-supported health and social services (for example, see [18]). This context makes the existence of an entity such as the MHF all the more important. Two key roles for the MHF in this regard are apparent: firstly, in rigorously documenting the challenges faced by non-nationals reliant on public healthcare in the city, and secondly, in providing evidence-informed responses to counter popular misconceptions that portray migrants as vectors of infectious diseases and as a burden on public healthcare resources [19]. Such efforts are extremely difficult to sustain in a context of shrinking funds for civil society, however, and where members work in grant-funded contexts where time is allocated according to funding. Yet it is hoped that members will continue to view the work of the MHF as sufficiently relevant to and supportive of their core mandates to justify their participation.

At the time of writing (September 2016), the MHF has developed a partnership with the Migration and Health Project Southern Africa (maHp) of the ACMS. This partnership builds on the long-standing involvement of the ACMS in the MHF, and aims to support the three focus areas of the MHF through the development of a renewed, coordinated response to migration and health in the city, involving research, dissemination and public engagement. Central to this response is a series of planned policy dialogues funded by maHp that will bring together civil society, academia and local and national government actors on issues relating to migration and health. The first dialogue – which explored how migration and mobility are affecting progress towards achieving the UNAIDS 90:90:90 targets [20] – was successfully held in August 2016 and included active participation from the City of Johannesburg and the South African National AIDS Council (SANAC). As a result of this dialogue, SANAC committed to involving the MHF in the development of a “migration-aware” response [11, 21] that emphasises the need for a local-level lens, in the next iteration of the National Strategic Plan (NSP) for HIV, STIs and TB.

Our findings suggest that a structure such as the Johannesburg MHF – including through its new partnership with maHp – has a vital role to play in crafting effective local responses to migration and health in a context of continued, and increasing, global, regional and national migration, and against the backdrop of rising anti-immigrant sentiments.

## Abbreviations

ACMS: African Centre for Migration & Society
HIV: Human Immunodeficiency Virus
IOM: International Organisation for Migration
MHF: Migrant Health Forum
NSP: National Strategic Plan
SANAC: South African National AIDS Council
STI: Sexually Transmitted Infection
TB: Tuberculosis
Wits RHI: Wits Reproductive Health and HIV Institute
